# Are we there yet? 40 years of successes and challenges for children and adolescents living with HIV

**DOI:** 10.1002/jia2.25759

**Published:** 2021-06-07

**Authors:** Rachel C Vreeman, Natella Y Rakhmanina, Winstone M Nyandiko, Thanyawee Puthanakit, Rami Kantor

**Affiliations:** ^1^ Department of Global Health and Health System Design Icahn School of Medicine at Mount Sinai New York NY USA; ^2^ Arnhold Institute for Global Health New York NY USA; ^3^ Division of Infectious Diseases Children’s National Hospital Washington DC USA; ^4^ Department of Pediatrics The George Washington University School of Medicine and Health Sciences Washington DC USA; ^5^ Technical Strategies and Innovation Elizabeth Glaser Pediatric AIDS Foundation Washington DC USA; ^6^ Department of Child Health and Paediatrics School of Medicine College of Health Sciences Moi University Eldoret Kenya; ^7^ Academic Model Providing Access to Healthcare (AMPATH) Eldoret Kenya; ^8^ Division of Infectious Diseases Department of Pediatrics Faculty of Medicine Chulalongkorn University Bangkok Thailand; ^9^ Division of Infectious Diseases Alpert Medical School Brown University Providence RI USA

**Keywords:** HIV, paediatrics, children, adolescents, antiretroviral therapy, resistance

Forty years ago, the first adult HIV cases were published, with infant cases following within a year [1]. As a few of these then‐babies approach their 40th birthdays, both their growth and science’s growth tell dramatic stories. Antiretroviral therapy (ART) transformed HIV from a deadly infection into a chronic disease. Just as miraculous, an AIDS‐free generation became imaginable, using ART to prevent >95% of perinatal transmission. While these advances in HIV prevention and treatment deserve celebration, attention should be devoted to remaining hurdles – such as behavioural, social viral suppression and drug resistance challenges – that must still be overcome to ensure successful life‐long outcomes for the global population of children and adolescents who have grown up with HIV (CAWH).

The global HIV impact for CAWH continues to be enormous: in 2020, 1.8 million children under 14 live with HIV, and every day 400 still acquire HIV and 270 die from it. Although ART access has expanded, only 53% of CAWH were receiving ART in 2019. Many countries do not screen mothers or infants for HIV, which leads to perinatal transmission, late childhood diagnosis, and deaths. Two million adolescents live with HIV globally, approximately 80% in sub‐Saharan Africa, for whom HIV remains the top cause of death. Older adolescents (15 to 19 years) are the only age group in which HIV‐related deaths are not decreasing. In the face of these young deaths, current care models clearly are not working; of adolescents 10 to 19 years with HIV, only 43% engage in care, 31% are retained in care and a dismal 30% are virally suppressed [2]. Added challenges of COVID‐19 pandemic‐related disruptions on HIV testing and care remain to be fully quantified and understood for CAWH.

Behavioural and social challenges evolve throughout growth and development, from infants who spit out poorly palatable liquid medications like lopinavir/ritonavir; to school‐aged CAWH who question why they take medicines; to adolescents living in stigmatizing environments and struggling to establish autonomy and fit in. Such challenges, on top of the difficulty accepting an HIV diagnosis, impact ART adherence and clinic engagement, with risks for worsening morbidity and mortality. Both adherence support and psychosocial counselling are less accessible to families in low‐and‐middle‐income‐countries (LMIC) [3]. Parents and caregivers struggle with disclosure to their children, often waiting until adolescence to explain how and why they have HIV out of concerns for their reaction and fears that they may disclose the family HIV status to others. Disclosure delays can hurt adherence, as can wrestling with diagnosis acceptance and avoiding sensitive disclosures to friends and sexual partners [4]. CAWH may have behavioural and mental health challenges, associated with more risky sexual behaviours, substance use and non‐adherence [5]. CAWH are also at substantial risk of stigma and its deleterious effects. Challenges can be distinct from adults, like bullying, discrimination at or exclusion from schools, growing up with caregivers’ fears of stigma, internalizing stigma, violence and prosecution tied to sexual/gender orientation [6], and exacerbations of poverty, malnutrition and inequitable healthcare access [7]. While CAWH‐focused HIV services may offer individualized behavioural and social support, transitioning into adult care often negatively affects older youth as they “age out” of more child‐friendly paediatric health systems into larger clinics that lack such support services. This transition period can be a time of increased risks of poor adherence and viral outcomes [8].

Multiple factors contribute to challenges with viral suppression in paediatric HIV care, and CAWH do not achieve similar suppression levels as adults [9]. First, overall global roll‐out of routine viral load testing has been slow, complicated by reliance on plasma‐based specimens, and, particularly for CAWH, limitations such as phlebotomy expertise and insufficient evidence for optimal scheduling of routine monitoring. Second, the limited number of ART formulations appropriate for and available to children make regimen modifications difficult, especially in LMIC. Only a quarter of approved formulations have dosing guidelines and are approved for children <2 years old, and may still be unpalatable or complex to administer [10]. Third, CAWH are less likely to participate in new therapeutic clinical trials, and regulatory approvals and guidelines are typically slow to extend to paediatric populations. The amalgamation of limited VL monitoring, fewer ART options and poor adherence support contributes to undetected treatment failures, delayed ART switches and drug resistance accumulation [11].

Evaluating drug resistance across the lifespans of CAWH is, therefore, a particular concern. However, resistance testing as part of clinical care remains limited in LMIC, and its true burden is largely unknown [12]. The few available studies demonstrate extensive resistance to ART regimens commonly used in CAWH, whereas longitudinal outcomes data are limited to non‐existent [13, 14]. Even as newer medications with high barriers to resistance are rolled out for CAWH (e.g. second‐generation integrase strand transfer inhibitors), scale‐up challenges in the context of implementation, prior relevant ART exposures, and limited availability of formulations may still negatively affect the trajectory and impact of resistance [15]. Largely unknown aspects of resistance (e.g. archived mutations, minority resistance variants, co‐occurrence of mutations within genomes, alternative resistance mechanisms) may accompany CAWH into adulthood and become difficult to overcome in the future.

An AIDS‐free generation should be possible, but we are not there yet. HIV remains a burden, not only in LMIC, but also in resource‐rich settings like the United States, where systemic impacts of racism on healthcare access, poverty, incarceration, stigma, mental health, and substance use are exacerbated by disparities in perinatal and adolescent HIV transmission and treatment outcomes. As we honour our gains in HIV care delivery over the past 40 years, we must address the specific, nuanced and long‐term challenges of living with HIV for CAWH, and realize that they cannot be treated as “typical adults with HIV” as they mature and grow older. Until we transform care systems, treatment regimens, adherence and mental health support, viral and drug resistance monitoring and global policy, this generation will remain vulnerable to high HIV transmission, morbidity and mortality. Setting priorities to support CAWH requires greater attention to the long‐term implications of the unique challenges they have faced over the first decades of the HIV pandemic so that they can successfully reach the next chapters of their lives.

## Competing interests

The authors declared no potential conflicts of interest with respect to the research, authorship, and/or publication of this article.

## Authors’ contributions

All five authors (RV, NR, WN, TP and RK) meet the criteria for authorship and have made substantial contributions to various facets of the manuscript. This manuscript was initially conceptualized and written by RV and RK. All authors reviewed, edited and contributed significantly to writing subsequent versions of the manuscript. All authors read and approved the final manuscript.
